# Limb Skin Temperature as a Tool to Predict Orthostatic Instability

**DOI:** 10.3389/fphys.2018.01241

**Published:** 2018-09-05

**Authors:** Oliver Opatz, Michael Nordine, Helmut Habazettl, Bergita Ganse, Jan Petricek, Petr Dosel, Alexander Stahn, Mathias Steinach, Hanns-Christian Gunga, Martina A. Maggioni

**Affiliations:** ^1^Charité – Universitätsmedizin Berlin, Institute of Physiology, Center for Space Medicine and Extreme Environments Berlin, Berlin, Germany; ^2^German Aerospace Center (DLR- Deutsches Zentrum für Luft- und Raumfahrt), Institute of Aerospace Medicine (Institut für Luft- und Raumfahrtmedizin), Cologne, Germany; ^3^Institute of Aviation Medicine, Military University Hospital Prague, Prague, Czechia; ^4^Division of Sleep and Chronobiology, Department of Psychiatry, Perelman School of Medicine at the University of Pennsylvania, Philadelphia, PA, United States; ^5^Department of Biomedical Sciences for Health, Università degli Studi di Milano, Milan, Italy

**Keywords:** lower body negative pressure, skin temperature, blood pooling, acceleration, orthostatic hypotension

## Abstract

Orthostatic instability is one of the main consequences of weightlessness or gravity challenge and plays as well a crucial role in public health, being one of the most frequent disease of aging. Therefore, the assessment of effective countermeasures, or even the possibility to predict, and thus prevent orthostatic instability is of great importance. Heat stress affects orthostatic stability and may lead to impaired consciousness and decrease in cerebral perfusion, specifically during the exposure to G-forces. Conversely, peripheral cooling can prevent orthostatic intolerance – even in normothermic healthy subjects. Indicators of peripheral vasodilation, as elevated skin surface temperatures, may mirror blood decentralization and an increased risk of orthostatic instability. Therefore, the aim of this study was to quantify orthostatic instability risk, by assessing in 20 fighter jet pilot candidates’ cutaneous limb temperatures, with respect to the occurrence of G-force-induced almost loss of consciousness (ALOC), before and during exposure to a push-pull maneuver, i.e., head-down tilt, combined with lower body negative pressure. Peripheral skin temperatures from the upper and lower (both proximal and distal) extremities and core body temperature via heat-flux approach (i.e., the Double Sensor), were continuously measured before and during the maneuver. The 55% of subjects that suffered an ALOC during the procedure had higher upper arm and thigh temperatures at baseline compared to the 45% that remained stable. No difference in baseline core body temperature and distal limbs (both upper and lower) skin temperatures were found between the two groups. Therefore, peripheral skin temperature data could be considered a predicting factor for ALOC, prior to rapid onset acceleration. Moreover, these findings could also find applications in patient care settings such as in intensive care units.

## Introduction

G-force (+Gz)-induced loss of consciousness (GLOC) occurs during exposure to strong forces of acceleration. The resulting stress on the cardiovascular system is caused by decentralization of blood to the lower limbs and the splanchnic vessels of the pelvis. Consequently, the cardiac preload is reduced and perfusion of the central nervous system decreases (Self et al., 1996). Similar to rapid changes in posture, that cause an orthostatic reaction, these forces can lead to loss of consciousness and postural tone. However, some individuals exhibit a high degree of cardiovascular resilience to +Gz, and therefore have greater orthostatic tolerance than others. The detailed mechanism and the contributors to orthostatic tolerance have yet to be elucidated, however, Yang et al. (2012) demonstrated that inter-individual differences in sympathetic firing pattern could be involved in orthostatic reactions during lower body negative pressure (LBNP). Several prodromal states have been described to be pathognomonic for orthostatic intolerance including dizziness, hearing loss, nausea, discomfort, sweating, palpitations, and “grayout” and “blackout” events (Benni et al., 2003). Regarding the exposure to high accelerations during space-flight take-off/landing or rapid in-flight maneuvers, orthostatic intolerance could lead to severe consequences, including a critical mission-compromising event. Therefore, a physiological parameter that would yield predictive information prior to the occurrence of an orthostatic event, would be invaluable, not only in aerospace medicine (Tripp et al., 2009), but also in clinical settings (Krakow et al., 2000). Current means to test for orthostatic intolerance – and GLOC-susceptibility are the tilt table, LBNP human centrifuge, and parabolic flight. Normally, cardiovascular parameters such as heart rate and non-invasive blood pressure are analyzed during these training methods (Durand et al., 2004). However, these values can show high inter-individual variation and there can be significant fluctuations in signal quality during high accelerations (Hanousek et al., 1997), which would provide unreliable and invalid physiological data. Further evidence by Simonson et al. (2003) showed that baseline heart rate and blood pressure demonstrated no significant differences amongst subjects with high versus low +Gz tolerance during LBNP testing. Thus, baseline hemodynamics seem to be unsuitable for predicting GLOC. However, Crandall et al. (2010) established that peripheral blood volume distribution caused by heat stress can reduce cerebral blood velocity. This distribution can thus be counted as an important factor in individual susceptibility to orthostatic events (Wilson et al., 2006). Earlier Crandall’s group found that external cooling may also improve orthostatic stability in normothermic subjects (Durand et al., 2004). In this light, we propose here a novel approach to test for GLOC-susceptibility using baseline proximal and distal skin temperature of the limbs monitored prior to a gravitational challenge. We hypothesize that subjects displaying a higher limb skin temperature (Tskin) prior to push-pull maneuver, i.e., head-down tilt (HDT) followed by LBNP, would be more prone to GLOC.

## Materials and Methods

### Subjects

Twenty male subjects, with a mean age of 28.2 ± 9.5 years, were tested by means of a standardized HDT/LBNP protocol and gave their written and informed consent before testing. As all participants were military pilots, the study was undertaken in compliance with the guidelines of the local Medical Military Advisory Board, which allowed the study and the use of the collected data. Mean anthropometric data for the subjects were the following: height 181.6 ± 6.7 cm, weight 80.2 kg ± 8.6, and BMI 24.3 ± 1.8 kg/m^2^. Each test subject was accustomed to daily high-workload endurance training. None had any cardiovascular, metabolic, or musculoskeletal pathologies. Exclusion factors were any history of syncope, arrhythmia, or other similar medical abnormalities. Each subject underwent a pre-test screening conducted by the flight physician. This included a physical exam and a resting 12-lead electrocardiogram. No reasons to exclude any subjects were found.

### Experimental Procedure

**FIGURE 1:**
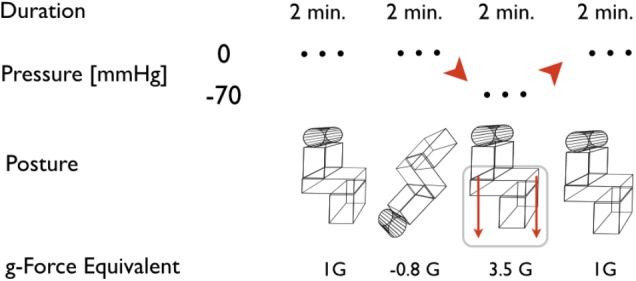
Scheme of the head-down tilt and lower body negative pressure (HDT/LBNP) testing. The depicted phases are assigned to posture of the subject and pressure in the LBNP. Acceleration equivalents are related to cardiovascular reaction at comparable accelerations during flight or long-arm centrifuge runs. Duration relates to the time the subjects were actually exposed to the referred posture and pressure at a steady level, excluding transition phases.

To test for orthostatic fitness, the combined HDT/LBNP test is used as a routine part of the Czech Air Force Centre of Flight Training (CLV) program. HDT/LBNP testing has been standardized for use at the Institute of Aviation Medicine (Dosel et al., 1998, 2007). The rapid change from the HDT to the head-up tilt with LBNP is known as the “push-pull maneuver” (Banks et al., 1994). LBNP followed a standardized phase profile which consists of four phases, as demonstrated in **Figure 1**. During baseline, subjects remained in an upright sitting position (Sitting); then, subjects were tilted head-down (HDT) for 2 min, then tilted back to head-up sitting position (Sitting) and simultaneously exposed to a negative pressure of -70 mmHg (onset at 2 s) for 2 min (Sitting/LBNP). Finally, the subjects were returned to baseline conditions without LBNP (Sitting). Upon entering the test facility, each test subject undressed down to their underwear and was fitted for instrumentation. Air temperature was kept constant at 27.0°C ± 0.6 with a humidity of 40–50%. No subject reported being cold or too warm during the experiment, nor exhibit any shivering or sweating prior to testing. Temperatures inside the LBNP chamber at baseline ranged with a median of 26.3°C between 24.2 and 27.2°C. Test subjects were placed in the LBNP device upright, seated position. A pressure seal was fitted around the participant at anterior superior iliac-level to ensure an air-tight seal. To monitor Tskin, a total of 5 thermometer probes were placed on each subject at various regions: (i) the anteromedial aspect of the left arm bisecting the biceps muscle, (ii) the dorsal side of the left hand 3 cm distal to the carpal region, (iii) the left anterior medial thigh, halfway between the anterior superior iliac crest and the patella region, (iv) 3 cm distal to the tarsal region of the leg, and (v) a thermistor attached to the dorsal aspect of the foot. Peripheral temperatures were measured via the Heally system (SpaceBit Gmbh, Wiesenick, Germany) using thermistors soldered to a silver-plated 32 American wire gauge copper wire (7/40) with polyurethane insulation. Core body temperature was measured using a double heat flux sensor (Gunga et al., 2008, 2009; Mendt et al., 2017), which was affixed to the medial forehead of each test subject. The influence of fluctuating air temperatures on the skin temperature probes was mitigated by ensuring a constant ambient temperature and environment. No side effects such as skin reactions to the applied electrodes were observed. Cardiovascular parameters such as heart rate (HR, bpm) and blood pressure (BP systolic/diastolic, mmHg) were continuously recorded non-invasively via Portapres monitoring (BMI-TNO, Amsterdam, Netherlands). Once all monitoring equipment was affixed to each subject and an uplink was established, an “all clear” signal was given by the flight physician and the phase profile exposure began. If any subject experienced syncope or cardiovascular instability during the study (such as a HR of <50 bpm, mean arterial pressure of <60 mmHg, or systolic BP of <90 mmHg – the so-called “stop criteria”), the flight physician could immediately terminate the LBNP exposure. In this case, the LBNP would be switched off and normal atmospheric pressure to the lower extremities would be re-established. These cardiovascular “stop criteria” are based on former investigations using LBNP and tilt tables (Hanousek et al., 1997). The experiment would also be interrupted immediately in case the subject described visual disturbances, such as blurred vision, tunnel vision, grayout, or blackout. In addition, the appearances of any autonomic symptoms such as sweating, nausea, or paresthesia would also halt the LBNP exposure.

### Data Analysis

Cardiovascular data were recorded continuously, with a resolution of 250 Hz for electrocardiography and a sample rate of 1 Hz as for BP data. Temperature data were also collected continuously at 1 Hz sample rate. For all variables, the values from the last 10 s of each 2 min phase (**Figure 1**) were averaged and taken into account for data analysis, in order to observe the maximum effect of each different posture/G level.

### Statistical Analysis

Data are reported as mean ± standard deviation (m ± SD), if not otherwise stated. Data were analyzed for normality via a Shapiro–Wilks test using SPSS (IBM Corporation, Armonk, NY, United States). Statistical comparisons were made using the more conservative Mann–Whitney *U*-test due to the non-normality of all data groups. Significance was defined as a *p* < 0.05. Data are reported using Tukey boxplots. The box represents the interquartile range (IQR) including the median, and the whiskers represent 1.5 × IQR above the third or below the first quartile. To determine the optimal cut-off temperatures and cardiovascular values to predict GLOC, receiver operating characteristics (ROCs) analyses were performed (Hanley and McNeil, 1982). To define cutoff values, we used the Youden’s I to estimate the probability of an informed decision (Youden, 1950). The statistical power for this study was set at 80% to determine a difference in Tskin of ±0.7°C for a minimum of 20 subjects.

## Results

**FIGURE 2:**
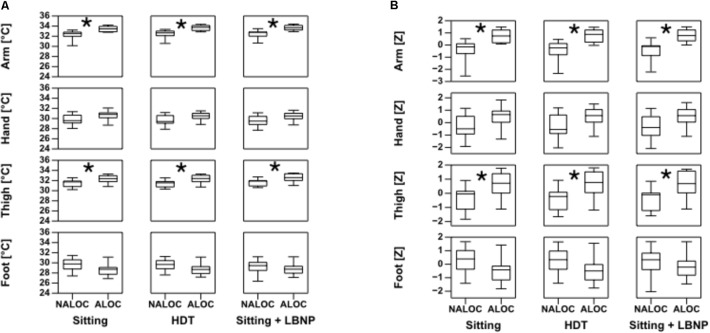
Boxplots of absolute limb temperature data on the left **(A)** and standard deviations as *Z*-values on the right **(B)**, in ALOC (i.e., almost loss of consciousness) versus NALOC (i.e., absence of almost loss of consciousness) subjects at baseline in the last 30 s while sitting, during head down tilt (HDT) and during sitting + LBNP (i.e., low body negative pressure). Significant differences (*p* < 0.05) found using the Mann–Whitney *U*-test are denoted by ^∗^.

Of the 20 subjects, 11 (55%) exhibited an almost loss of consciousness (ALOC), leading to test termination by the flight physician. In all cases the reason for this was a strong decrease in systolic BP. Therefore, subjects were classified into two groups, those who suffered an ALOC and those who did not (NALOC), to allow a comparison. Overall the intervention induced similar changes in Tskin for all subjects (variation from 0.2 to 0.5°C). Core body temperature did not exhibit fluctuations nor differences between ALOC/NALOC subjects. At baseline, mean core body temperature of all subjects was 36.9 ± 0.4°C. Statistically significant baseline differences between ALOC and NALOC groups were found regarding Tskin only in the upper arm and thigh. Baseline Tskin at the upper arm in the NALOC group was 32.03 ± 1.08°C as compared to 33.23 ± 0.66°C in the ALOC group (*p* = 0.04). Baseline Tskin at the thigh was 31.34 ± 0.81°C in the NALOC group as compared to 32.24 ± 0.77°C in the ALOC group (*p* = 0.006). These values are displayed as boxplots in **Figure 2**. At baseline, diastolic BP was slightly higher in the NALOC group (*p* = 0.056). Statistical comparisons at baseline of core body temperature (*p* = 0.45), HR (*p* = 0.86), systolic BP (*p* = 0.32), and distal limb Tskin temperatures revealed also no significant differences between groups (as for foot *p* = 0.10 and as for hand *p* = 0.13).

**FIGURE 3:**
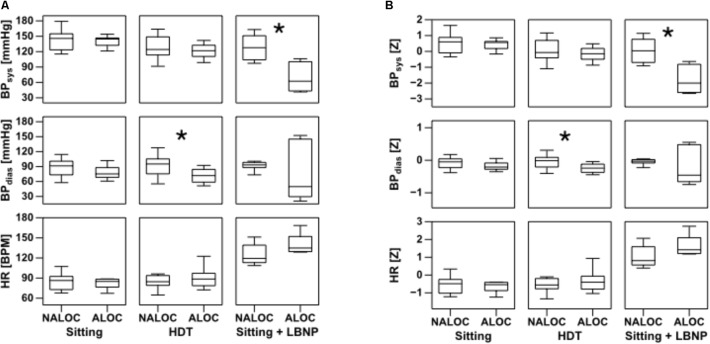
Boxplots of absolute values of HR, diastolic (BPdias) and systolic (BPsys) blood pressure on the left **(A)** and its standard deviations as *Z*-values on the right **(B)** in the last 30 s while sitting, during head down tilt (HDT) and during sitting + LBNP (i.e., low body negative pressure). Significant differences (*p* < 0.05) found using the Mann–Whitney *U*-test are denoted by ^∗^. ALOC, almost loss of consciousness; NALOC, absence of almost loss of consciousness (see also text for abbreviations).

During HDT, Tskin showed significant differences of upper arm and thigh between ALOC and NALOC group, i.e., NALOC exhibited significantly lower temperatures than ALOC subjects (*p* = 0.002 and *p* = 0.016, respectively) (**Figure 2**). Moreover, ALOC subjects exhibited a significantly lower diastolic BP than NALOC subjects (*p* = 0.004) (**Figure 3**).

During Sitting/LBNP, a significant increase of upper arm and thigh Tskin was found in the ALOC group with respect to NALOC (*p* = 0.002 and *p* = 0.01, respectively, see **Figure 2**). Furthermore, during this phase, a significant decrease in systolic BP was retrieved as for ALOC with respect to NALOC subjects (*p* = 0.007), whereas diastolic BP was similar between groups (*p* = 0.60); HR showed no difference between the groups (*p* = 0.54 in HDT and *p* = 0.18 in Sitting/LBNP, see **Figure 3**).

**FIGURE 4:**
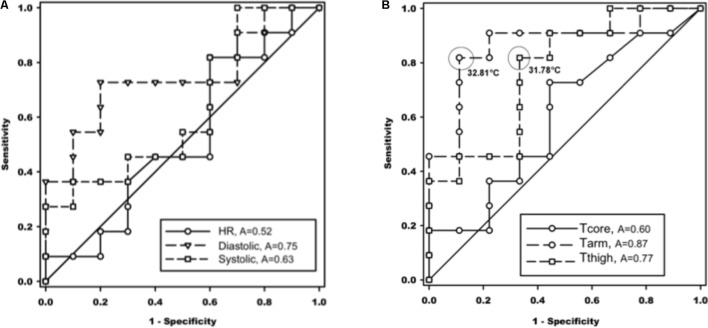
Receiver operating characteristic (ROC) curves of association of cardiovascular values in the left panel **(A)**, and temperatures in the right panel **(B)**, with almost loss of consciousness (ALOC) occurrence. The cut-off values were selected at maximum sensitivity + specificity and highlighted in circles. A, area under the curve; HR, heart rate (*p* = 0.86); Diastolic, diastolic blood pressure; Systolic, systolic blood pressure; Tcore, core body temperature; Tarm, upper arm temperature; Tthigh, thigh temperature (see also text for abbreviations).

The ROC was used to show the diagnostic ability of the method and the discrimination thresholds. It revealed significant associations between cutaneous arm and thigh temperatures with ALOC. Optimal cut-offs to predict ALOC were 32.81°C for arm temperature and 31.78°C for thigh temperature (**Figure 4B**).

### Limitations

So far, results of this study could only be applied for a limited specific population groups (young healthy men, already trained as jet pilots), therefore further studies are needed to cover more individual parameters such as gender, age and a spectrum of anthropometric differences. In this context it is needed to study a greater number of subjects grouped by the described parameters to exactly define the efficacy of the method.

## Discussion

The results of this study showed a link between individuals’ proximal limb Tskin and orthostatic stability during a simulation of aerospace braking maneuvers in a temperature-controlled environment (i.e., push-pull maneuver).

Other research has focused on the effects of external temperature regarding orthostatic stability during LBNP. It has been shown that orthostatic tolerance in men is decreased during heat stress (Kirsch et al., 1986; Rubinstein and Sessler, 1990; Robertson, 2008; Schlader et al., 2015). Moreover, whole-body heating during LBNP leads to a decrease in central venous pressure as well as pulmonary capillary wedge pressure (Wilson et al., 2007). Furthermore, the link between increases in skin blood flow during hyperthermia and orthostatic tolerance has been extensively studied by the group of Craig Crandall (Shibasaki et al., 2006; Wilson et al., 2007; Crandall et al., 2010). On the contrary, cooling of the periphery, and thus the simultaneous decrease in skin blood flow, does appear to have a positive impact on orthostatic tolerance in a normothermic person. Durand, indeed found that active skin cooling of the normothermic subject might improve orthostatic stability (Durand et al., 2004). In his study, environmental temperature was kept constant in order to minimize the effect of ambient temperature on peripheral vasomotor activity, demonstrating that, despite thermo-neutral ambient temperature conditions, Tskin does influence orthostatic stability. This is in concordance with our original reasoning that Tskin would correlate with cutaneous vasomotor activity and that higher temperatures would indicate increased perfusion to the periphery, thus depriving the central circuit of volume. However, this would need to be verified by direct measurement of vasomotor activity via near-infrared spectroscopy (NIRS), to concretely solidify the link between the two factors. During acceleration conditions (i.e., LBNP), a decrease in blood pressure activates the baroreceptor reflex which induces strong sympathetic activation (Convertino et al., 2004). This sympathetic counter-response results in adrenergic α-receptor-mediated vasoconstriction as well as β1-receptor mediated HR increase. Maintenance of BP during orthostatic stress depends on the careful shifting of blood flow from non-essential organ systems to essential systems. The increased vasomotor activity plays a key role in maintaining mean arterial pressure via increases in arteriolar tone, which in turn increases systemic vascular resistance (Hachiya et al., 2010), while at the same time inducing a decrease in cutaneous perfusion. As previously reported by Rubinstein and Sessler (1990), it was shown that increased vasoconstriction in the fingertip had a significant negative correlation with skin surface temperature. In contrast, a study by Cotie et al. (2011) showed that cutaneous temperature changes did not reflect changes in femoral artery blood flow. The authors, however, conceded that bulk blood flow measurements may not be sensitive enough to reflect changes in cutaneous microvascular perfusion.

In our study, we exploited Rubinstein’s findings and measured skin surface temperature at the limbs to estimate changes in skin perfusion via skin surface temperatures. As stated, our key findings concerning baseline Tskin during the HDT/LBNP procedure showed that proximal Tskin within the NALOC group were not significantly different from baseline, thus exhibiting no change in cutaneous perfusion across different phases of exposure. This trend was also seen in the ALOC subjects. It occurs when the central nervous system fails to receive adequate perfusion, resulting in a syncopal reaction (Wilson et al., 2006). The monitoring of brain perfusion during simulated hyper-gravity can be performed very effectively using NIRS, as published by Ryoo et al. (2004). While reduction of brain perfusion is the most important resulting factor, peripheral blood volume distribution might be the most important predicting factor of subject’s G-tolerance. Thus, we can only observe the effect, but not the cause, which is known to be venous blood pooling in the splanchnic compartment and lower limbs (Blaber et al., 2013) and insufficient cutaneous vasoconstriction to maintain blood pressure (Crandall et al., 2010). According to these findings the predictive peripheral temperature values during LBNP were determined using ROC curves. It could be demonstrated that heart rate and systolic blood pressure underperformed as predictive values. We hypothesized that lower baseline vessel tone, as reflected by higher Tskin, may also indicate lower ability to increase cutaneous vascular resistance during orthostatic challenges. Our results were able to confirm this finding – at least, amongst a small subject pool such as this one. Thus, peripheral Tskin data could be employed as a predicting factor for ALOC/NALOC prior to rapid onset acceleration. Significant differences were detected in baseline upper arm and thigh temperatures between the ALOC and NALOC groups (see **Figure 4**). Both temperatures predicted ALOC with a sensitivity of >0.8 and, in case of the upper arm temperature, with a specificity of >85%. The reason why only proximal and not distal limb temperatures were associated with ALOC may be due to the larger proximal density of blood vessels, different innervation of the proximal and distal limbs, and different receptor density. Such differences were also reflected by the considerably lower baseline temperatures at the distant limb site (i.e., an anatomical bordering effect). Other scenarios involve the variability of autonomic innervation of the proximal and distal limbs as well as different receptor density (Schiller, 2003). Due to the larger bulk of vasculature in the proximal limbs versus distal limbs, this could lead to a discrepancy with regards to autonomic vasculature innervation. Such differences were reflected by the considerably lower baseline temperatures at the distant limb site. Thomas (2011) reported that increases in vasomotor tone of the capacitance vessels would decrease tissue blood volume while, at the same time, increasing cardiac preload.

However, further studies on the concrete mapping of the distribution of autonomic fibers in the limbs are needed to confirm this. At baseline, there were no significant differences among subjects with respect to cardiovascular parameters. There was a trend in the ALOC subjects to exhibit lower diastolic blood pressure compared to NALOC subjects. Diastolic pressure is an indicator of vascular resistance (Lacolley et al., 1992) and test subjects tending to ALOC exhibited lower diastolic pressure, which would equate to lower vascular resistance. Thus, this is compatible with the higher Tskin in subjects prone to ALOC.

According to van Genderen et al. (2013) it could also be shown that the Pulse-Perfusion-Index (PPI) could be used to detect hypovolemia before it becomes clinically apparent. In contrast to our study van Genderen et al. (2013) used a stepwise descending LBNP pressure scenario where they found a decrease in PPI already at a pressure of -20 mmHg. However, the reduction of PPI prior to LBNP exposition as a predictor of syncopal events could only be shown at a single subject collapsed in the LBNP.

The measuring of peripheral temperatures could therefore be a more sensitive early indicator of vasomotor activity than the measurement of cardiovascular values alone. Once established as a sensitive and reliable tool, this method, coupled with classical cardiovascular monitoring, can be employed in real world military high G-force (or +Gz) maneuvers, Earth to orbit manned space missions, as well as civilian air transport. This technique is non-invasive, sensitive, and easy to employ. When combined with classical cardiovascular monitoring, it adds a new individual method to monitor volume distribution during orthostatic stress.

## Ethics Statement

All participants were military pilots, therefore the study was undertaken in compliance with the guidelines of the Czech Aviation Medicine Military Advisory Borad (Prague), which approved the protocol, allowed the study and the use of collected data. All subjects gave written informed consent in accordance with the Declaration of Helsinki.

## Author Contributions

OO conceived the study and wrote the manuscript with MM. MN contributed to drafting the manuscript and to statistics. BG, JP, and PD performed data collection and data analysis. AS, H-CG, and MM contributed to the study design, and with HH and MS provided expertise and feedback. OO formatted, and with assistance of MM, revised manuscript.

## Conflict of Interest Statement

The authors declare that the research was conducted in the absence of any commercial or financial relationships that could be construed as a potential conflict of interest.
